# Gallic and Ellagic Acids Differentially Affect Microbial Community Structures and Methane Emission When Using a Rumen Simulation Technique

**DOI:** 10.1021/acs.jafc.4c06214

**Published:** 2024-11-26

**Authors:** Michele Manoni, Florian Gschwend, Sergej Amelchanka, Melissa Terranova, Luciano Pinotti, Franco Widmer, Paolo Silacci, Marco Tretola

**Affiliations:** † Department of Veterinary Medicine and Animal Science, University of Milan, Via dell’Università 6, Lodi 26900 Italy; ‡ Molecular Ecology, Agroscope, Zurich 8046, Switzerland; § AgroVet-Strickhof, ETH Zurich, Lindau 8315, Switzerland; ∥ CRC Innovation For Well-Being And Environment (I-WE), University of Milan, Milan, 20134 Italy; ⊥ Paolo Silacci − Animal Biology, Agroscope, Posieux 1725, Switzerland; # Swine Research Group, Agroscope, Posieux 1725, Switzerland

**Keywords:** tannins, ruminants, Rusitec, urolithins, microbiota

## Abstract

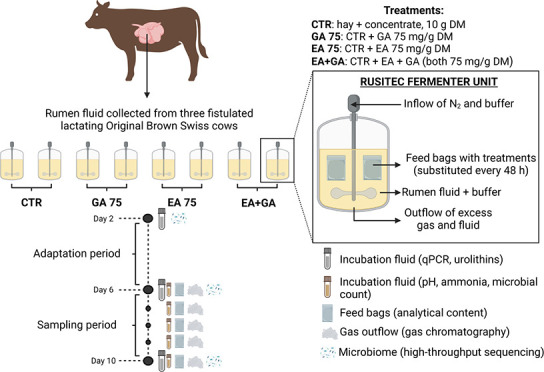

Dietary tannins can affect rumen microbiota and enteric fermentation to mitigate methane emissions, although such effects have not yet been fully elucidated. We tested two subunits of hydrolyzable tannins named gallic acid (GA) and ellagic acid (EA), alone (75 mg/g DM each) or combined (150 mg/g DM in total), using the Rusitec system. EA and EA+GA treatments decreased methane production, volatile fatty acids, nutrient degradation, relative abundance of *Butyrivibrio fibrisolvens*, *Fibrobacter succinogenes*, *Ruminococcus flavefaciens* but increased *Selenomonas ruminantium*. EA and EA+GA increased urolithins A and B. Also, EA and EA+GA reduced bacterial richness, with limited effects on archaeal richness. For bacteria, *Megasphaera elsdenii* was more abundant after EA and EA+GA, while Methanomethylophilaceae dominated archaea in all treatments. EA was more effective than GA in altering rumen microbiota and fermentation but GA did not reduce VFA and nutrient degradation. Thus, dietary supplementation of EA-plant extracts for ruminants may be considered to mitigate enteric methane, although a suitable dosage must be ensured to minimize the negative effects on fermentation.

## Introduction

Ruminants convert low-quality plant fibers into energy and proteins through the enteric fermentation processes exerted by the rumen microbiota. Enteric fermentation leads to the production of volatile fatty acids (VFA), the major source of energy for ruminants, but it also causes the emission of gases such as methane (CH_4_), carbon dioxide (CO_2_), and hydrogen (H_2_).^1,2^ Over the past 40 years, atmospheric concentration of the greenhouse gases (GHG) CH_4_ and CO_2_ has increased by 18% and 23% respectively,^3^ with similar contributions from natural and anthropogenic sources.^4^ The CH_4_ has a shorter atmospheric lifespan^5^ and a higher global warming potential than CO_2_ because of its higher radiative forcing.^6^ Livestock-related CH_4_ emissions have grown 4-fold over the past 130 years, currently accounting for a third of global anthropogenic CH_4_ emissions.^7^ Enteric fermentation from ruminants is the main source of livestock-related CH_4_ emission, responsible for approximately 90% of livestock-related CH_4_ emissions. The remaining 10% comes from feed production and manure emissions.^8^ Livestock has a primary role in CH_4_ emissions, thus strategies to reduce CH_4_ emission are needed for a more sustainable livestock production. Since CH_4_ is a physiological product of rumen methanogenic archaea, the mitigating strategies should target CH_4_ production without impairing energy production and animal well-being.^9^ In this regard, a promising strategy is the use of tannins as dietary supplements for ruminants.^10^

Tannins are secondary plant metabolites able to bind feed proteins,^11^ subtracting them from the microbial degradation occurring in rumen. Tannins are known to affect CH_4_ production through direct targeting of methanogenic pathways of archaea or indirect affecting feed fermentation by other rumen microorganisms, such as bacteria and protozoa whose H_2_ production is essential for methanogenesis.^11^ If properly balanced, tannins can positively affect rumen fermentation by reducing CH_4_ emissions and ammonia (NH_3_) formation while avoiding detrimental effects on the fermentation activity of rumen microbiota.^12^ Tannins are classified as condensed tannins (CTs) and hydrolyzable tannins (HTs). Both classes are able to reduce *in vitro* CH_4_ production through different mechanisms based on their molecular weights, because CTs have a higher molecular weight and ability to bind macromolecules than HTs.^13^ CTs are able to reduce CH_4_ by decreasing fiber fermentation in rumen, whereas HTs are less able to bind macromolecules but can reach CH_4_ reduction by more easily targeting the action of archaea methanogens, even though HTs are more degradable in rumen.^10,14^ Tannin-containing plant extracts were applied *in vitro* to observe the effect on rumen fermentation.^15,16^ Costa et al.^17^ observed that CTs from mimosa exerted a stronger reducing effect than HTs from chestnut on VFA and a selected set of rumen bacteria. However, the mode of action of individual tannin subunits alone or combined on rumen fermentation has not been fully elucidated.

To address this, we applied Rusitec, a well-established, continuous, and standardized *in vitro* system to simulate rumen fermentation for several days (e.g., 10 days) while maintaining fermentation parameters over time,^18^ thus allowing for a better characterization of the persistence and consistence of the effects investigated.^19^ When coupled with high-throughput sequencing, Rusitec provides detailed insights about the alteration of the rumen microbial communities following different treatments. Examples of tested treatments are the use of a pathogenic *C. perfringens* strain,^20^ choline,^21^ and a blend of essential oils and tannin-rich plant extracts.^22^ In addition, rumen microorganisms can degrade HTs and produce secondary tannin metabolites like urolithins, which result from the metabolism of ellagitannins, of which ellagic acid (EA) is a subunit. Urolithins are considered the executors of the action usually ascribed to ellagitannins in rumen, but there is still no clear evidence.^23−25^

In a previous study, we tested the effect of EA and gallic acid (GA) on rumen fermentation using the Hohenheim Gas Test.^26^ EA and GA are classified as phenolic acids and are the subunits of the more complex HTs.^25^ The treatments with EA alone (75 mg/g DM) or in combination with GA (both at 75 mg/g DM) reduced CH_4_ production per unit of dietary DM, digestible OM (dOM), CO_2_, and VFA, as well as NH_3_ formation, more than GA alone. However, EA also lowered nutrient degradation in rumen. The aim of this study was to investigate the effect of EA and GA using a 10-day Rusitec system by focusing on the: (i) influence of tannins on rumen total gas production, CH_4_, NH_3_, VFA, and nutrient degradation, (ii) kinetics of urolithin A (UroA) and urolithin B (UroB) production, (iii) changes in the community structures of rumen bacteria and archaea, and (iv) correlation between gas production and relative abundance of selected groups of rumen microorganisms.

## Materials and Methods

### Experimental Design, Reagents, and Incubated Materials

The Rusitec system (for a detailed description, see ref (27)) was used to incubate three treatments and one control in three consecutive experimental runs. Each run lasted for 10 days, with days 1 to 5 allowing the system to reach a steady-state condition, and days 6–10 sampling and data collection. A basal diet of ryegrass hay and barley concentrate (10 g dry matter (DM), 7.5:2.5 ratio) was added to each fermenter every day. The ryegrass hay was ground to pass through a 5 mm sieve, whereas the barley concentrate was ground to a particle size of 1 mm using a centrifugal mill (Model ZM 200, Retsch GmbH, Hann, Germany). The nutrient composition of the basal diet was (g/kg DM) 964 organic matter (OM), 36 ash, 109 crude protein (CP), 376 crude fiber (CF), 751 neutral detergent fiber (NDF), 417 acid detergent fiber (ADF), 13.9 acid detergent lignin (ADL) and 19 ether extracts (EE). Fiber fractions are expressed excluding residual ashes. The basal diet was used as the control substrate (CTR). For the treatments, CTR was supplemented with GA and EA, alone or in combination (EA+GA). GA and EA were purchased from Sigma-Aldrich (St. Louis, MO, US). The purity level was ≥98.5% for GA and ≥95% for EA. The four treatments were: (i) basal diet alone (CTR), (ii) EA-supplemented CTR (EA, 75 mg/g DM), (iii) GA-supplemented CTR (GA, 75 mg/g DM), and (iv) EA and GA-supplemented CTR (EA+GA, both at 75 mg/g DM for a total of 150 mg/g DM). Those dosages were chosen based on their ability to elicit significant microbial and metabolic responses in an *in vitro* setting, as already observed in a previous, short-term study.^26^ In addition to the four treatment groups (CTR, EA, GA and EA+GA), we categorized the treatments into EA+ (containing ellagic acid) and EA- (lacking ellagic acid) based on observations showing that GA had a low impact on fermentation traits. This distinction allowed for further investigation into the specific effects of EA on microbial community structures and gas emissions. These groupings were applied to all subsequent analyses of microbial and fermentation outcomes.

### Rumen Fluid Collection

Donor animals were kept according to the Swiss guidelines for animal welfare, and the rumen fluid collection procedure was approved by the Cantonal Veterinary Office of Zurich (approval number ZH113/18). Three runs were performed. Rumen fluid was collected before the morning feeding from one animal per run, for a total of three rumen cannulated lactating Original Brown Swiss cows. Donor cows were fed 17.8 kg DM/day of a total mixed ration (TMR) composed of (% DM) grass silage (48%), maize silage (20%), sugar beet pulp (17%), hay (8%), concentrate (8%), and mineral supplement (0.2%). The pH of the fresh rumen fluids ranged between 5.9 and 6.7. Preheated glass bottles with water at 39 °C were used to keep the rumen fluid warm during transport. The inoculation took place within 2 h after rumen fluid collection. The rumen fluid was then filtered through four layers of medicinal gauze (pore size 1 mm) before being transferred into the fermenters.

### Operation of the Rusitec

The Rusitec consisted of eight 1-L fermenters. At the beginning of each run, the fermenters were filled with 800 mL of strained rumen fluid and 100 mL of artificial saliva composed of 9.80 g/L NaHCO_3_, 4.67 g/L Na_2_HPO_4_ × 2H_2_O, 0.47 g/L NaCl, 0.57 g/L KCl, 0.05 G/L CaCl_2_ × 2H_2_O, and 0.13 g/L MgCl_2_ × 2H_2_O. The components of artificial saliva were dissolved in distilled water. The fermenters were located in a heated water bath maintained at 39.5 °C. The incubation fluid was slowly moved up and down by an electric motor (six times per minute). The diet (CTR, GA, EA, or EA+GA) was added daily in nylon bags (70 × 140 mm, pore size 100 μm) to each fermenter. Each of the four treatments was allocated in duplicate to the eight fermenters in a completely randomized design. On day 1, two bags were incubated in each fermenter, one containing the respective experimental diet and one containing about 40 g of fresh matter of solid rumen content. On day 2, each fermenter was opened, and the bag with fresh matter was removed, squeezed, and washed in artificial saliva. The liquid fractions of the washings were returned to each fermenter, and the removed bag was then replaced with a new bag containing the experimental diet, for a total of two bags per fermenter. Each feed bag was incubated for 48 h and then substituted with a new one containing the same diet. Incubation of the substrate with GA and EA started on day 1 and lasted until day 10. Each fermenter was flushed with N_2_ gas for 3 min to maintain anaerobic conditions after the daily substitution of the feed bags. The flow of artificial saliva to the fermenters was continuous at about 400 mL per day, resulting in a dilution rate of the incubation fluid of about 40% per day. The overflow incubation fluid was collected in glass flasks and immediately frozen at −20 °C to stop fermentation. The volume of overflow from each fermenter was recorded to allow for adjusting the flow rate of artificial saliva in the fermenters in real time.

### Sample Collection and Laboratory Analyses

Feed samples were lyophilized (Delta 1-24 LSC, Christ, Osterode, Germany) and ground (Brabender mill, Brabender, Duisburg, Germany). DM and ash contents were measured gravimetrically by oven drying (prepASH 229, Precisa, Dietikon, Switzerland) for 3 h at 105 °C and subsequently incinerating at 550 °C for 4 h. The difference between DM and ash was defined as OM. The contents of the NDF and ADF (NDF: method ISO 16472:2006; ADF: ISO 13906:2008) were determined using Fibertherm (Gerhardt, Königswinter, Germany). The total N content of the feed samples was determined using the Kjeldahl method (AOAC International, 1995; method 988.05). To calculate the CP content, the N content was multiplied by 6.25. Ether extract content was analyzed by extraction following hydrolysis (ISO 6492:1999).

Every day, 3 h before replacing the feed bags, 10 mL of incubation fluid was collected directly from each fermenter. These samples were analyzed for pH, redox potential, and NH_3_ using a potentiometer (pH: model 913, Metrohm; redox: model 632, Metrohm; NH_3_: model 713, Metrohm, Herisau, Switzerland) equipped with the respective electrode. For VFA analysis, additional samples of 4 mL were collected and mixed with H_2_SO_4_ 50% (m/v) to stop fermentation. These samples were immediately frozen at −20 °C until HPLC determination, as described by Manoni et al.^26^ Furthermore, the samples were used for microbial counting under a microscope. Before counting, the samples were mixed with Hayem solution at 1:0.1 and 1:100 for protozoa and bacteria, respectively. Protozoa were counted using a Neubauer hemocytometer (0.1 mm depth, Blau-Brand, Wertheim, Germany), whereas bacteria using a Bürker hemocytometer (0.02 mm depth, Blau-Brand, Wertheim, Germany). On days 2, 6, and 10, two additional samples of 10 mL were collected from each fermenter and stored at −80 °C. One sample was used for DNA extraction, PCR, and high-throughput sequencing. The other sample was used to measure UroA and UroB, two secondary metabolites of ellagitannins produced by microbial fermentation.^25^ The sampling days were chosen to obtain a picture of the variation occurring in the microbial community and the related level of urolithins in the initial (day 2), middle (day 6), and final stage (day 10) of incubation.

Fermentation gas was collected in gas-tight aluminum bags (TECOBAG 8 L, PETP/AL/PE: 12/12/75 quality; Tesseraux Container GmbH, Bürstadt, Germany). Gas production was analyzed every day by collecting the gas samples from the bags and injecting them into a gas chromatograph (GC-TCD 6890 N, Agilent Technologies, Wilmington, NC, US), as described by Manoni et al.^26^ The total amount of gas produced was quantified using the water displacement technique.^27^ The amount of nitrogen gas injected was subtracted from the measured gas amount in the aluminum bag to obtain total gas production. The feed bags removed every 48 h from the fermenters were washed with cold water and without detergent in a domestic washing machine, squeezed, and stored at −20 °C. The feed bags were lyophilized for 48 h, allowed to air-dry for 24 h, and weighed. Later, the feed residues contained in the bags from day 6 to day 10 were mixed, ground to pass a 0.5 mm sieve, and analyzed for their analytical contents, as previously described for the feed samples. The analytical composition was then used to determine the degradation of the feed components, calculated as the amount of material that disappeared from the feed bag after 48 h of incubation. The apparent degradation was expressed in percentage as the ratio of g degraded/g incubated feed.

### Measurement of Urolithin A and Urolithin B

#### Sample Extraction

All chemicals, reagents, as well as UroA and UroB standards, were of analytical grade purchased from Merck (Darmstadt, Germany). The samples were extracted according to the protocol of Garcia-Villalba et al.^28^ Briefly, an aliquot of 2 mL of incubation fluid was mixed with 5 mL of ethyl acetate acidified with 1.5% formic acid. The mixture was vortexed for 2 min and centrifuged at 2500 g for 10 min. The organic phase was separated and evaporated by a vacuum rotary evaporator (Heidolph, Schwabach, Germany) at 35 °C. The dry sample was then redissolved in 200 μL of 0.1% formic acid in water: methanol (90:10) and 5 μL of 100 μg mL^–1^ of internal standard (6,7-dihydroxycoumarin) was added. The extract was then diluted 100 times before injection into the UPLC-HRMS system.

#### UPLC-HRMS Analysis

The analysis was carried out by a UPLC-HRMS system made by a Vanquish device (Thermo Fisher Scientific, Waltham, MA, US) coupled to a Thermo Orbitrap Exploris 120 (Thermo Fisher Scientific, Waltham, MA, US) equipped with a heated electrospray ionization (HESI) source. A Raptor ARC-18 5 μm, 150 × 2.1 mm column (Restek, Bellefonte, PA, US) was used for the chromatographic separation. Mobile phases A (0.1% aqueous formic acid) and B (MeOH) were mixed during the gradient, which started with 5% B kept for 1 min, increasing to 95% in 7 min and remaining until the 11th min. After 0.5 min, the initial conditions were reestablished until the 15th min. The flow was set at 0.3 mL min^–1^. Regarding the detector, the capillary and vaporizer temperatures were set at 330 and 280 °C, respectively, the sheath and auxiliary gas at 35 and 15 arbitrary units (AU), and the electrospray voltage at 3.50 kV in negative mode. Full-scan (FS) acquisition was combined with the parallel reaction monitoring (PRM) mode for the confirmatory response based on an inclusion list. The FS worked with a resolution of 60,000 fwhm, a scan range of 150–400 *m*/*z*, a standard automatic gain control (AGC), an RF lens of 70%, and an automatic maximum injection time. The PRM acquisition operated at 15,000 fwhm, with a standard AGC target, an automatic maximum injection time and scan range mode, and an isolation window of 1 *m*/*z*. Fragmentation of the precursors was optimized with a two-step normalized collision energy (40 and 60 eV). The precursor of UroA was the ion at 227.0350 *m*/*z*, and that of UroB was at 211.0401 *m*/*z*; their main fragments were at 159.0449 and 167.0501 *m*/*z*, respectively. The software used was XcaliburTM 4.5 (Thermo Fisher Scientific, Waltham, MA, US). The limit of quantification was 5 ng mL^–1^.

#### Quantitative PCR

Seven selected microbial groups and species representing the major rumen bacteria involved in the fermentation of dietary polysaccharides and the major methanogenic archaea were explored using quantitative PCR (qPCR).^29,30^ The DNA was extracted using a QIAMP Fast DNA Stool Mini Kit (Qiagen, Hombrechtikon, Switzerland), following the method reported in Böttger et al.^31^ Briefly, 2 mL of incubation fluid samples were centrifuged at 6500 RCF for 30 min at 4 °C to collect and resuspend the pellet using the Inhibitex buffer. This solution was heated at 90 °C for 5 min. After vortexing and centrifuging at 16,000 RCF for 1 min, the concentration of the DNA extracts was measured with a Qubit4 fluorometer (Thermo Fisher Scientific, Waltham, MA, US), and the quality of the DNA extracts was measured with a QSep100 device (Bioptic, New Taipei City, Taiwan). The DNA extracts were diluted to a final concentration of 4 ng/μL and then real-time qPCR was performed as reported in detail by Manoni et al.^26^ The relative abundance was measured in relation to the abundance of the total bacterial 16S rDNA, used as the reference sample, and measured by amplification with the 16v3 primers, as previously described.^32,33^

#### High-Throughput Sequencing

DNA extracts of fresh rumen fluids, as well as of samples from days 2, 6, and 10, were used for the assessment of bacterial and archaeal communities using high-throughput sequencing. Variable regions 3 and 4 of the rRNA gene sequence were amplified using primers 341F (5′-CCTAYGGGDBGCWSCAG-3′) and 806R (5′-GGACTACNVGGGTHTCTAAT-3′)^34^ for bacteria, and Arch349F (5′- GYGCASCAGKCGMGAAW-3′) and SSU666ArR (5′-HGCYTTCGCCACHGGTRG-3′)^35^ for archaea at a concentration of 1 μM in PCR reactions of 25 μL. TruSeq adapter sequences were appended to the primers to allow for subsequent sequencing library construction. Three PCR reactions, each with 12 ng of DNA, were performed for all samples and the two markers. PCR consisted of an initial denaturation step at 95 °C for 2 min, followed by cycles of denaturation at 94 °C for 40 s, annealing of bacteria or archaea PCR primer pairs at 56 or 66 °C for 40 s, and an elongation step at 72 °C for 1 min. A final elongation step was performed at 72 °C for 10 min. Amplifications were achieved with 25 cycles for the bacterial markers and 40 cycles for the archaeal markers. The triplicate PCR products were pooled prior to sequencing. Library preparation and NextSeq Illumina sequencing were performed at the Functional Genomics Centre of the University of Zurich. Raw amplicon sequences were quality filtered and grouped into amplicon sequence variants (ASVs) using a pipeline largely based on vsearch,^36^ which included several filtering steps, such as primer pruning, removal of sequences with a maximum expected error greater than 1, chimera removal, and target verification using Metaxa version 2.2.3.^37^ For the taxonomic assignment, ASVs were compared to release 214 of the genome taxonomy database (GTDB)^38^ using an Ribosomal Database Project (RDP) classifier implemented in mothur version 1.47.0.^39^ Sequences that did not represent targets, that is, nonbacterial or nonarchaeal sequences, including chloroplast or mitochondrial sequences identified using the SILVA database version 138 as a reference, were removed prior to further analyses.

#### Statistical Analysis

The significances of treatments, time (day) and their interaction were analyzed by repeated measures ANOVA using linear mixed-effects regression models (Lmer) implemented in Rstudio (version 4.0.5). All models contained the treatment and the day as fixed effects, while the run and fermenters were considered random effects. Because only one value per fermenter is obtained for nutrient degradation data, only the treatment was considered as a fixed effect for the statistics of such data. For pairwise comparisons, a modified Tukey test for multiple comparisons of means, the Sidak function, was performed. Statistical means and standard error of the means (SEM) were calculated with the lsmeans function from the package emmeans. The residuals of the Lmer models were checked for normality and homoscedasticity. If those conditions were not respected, the independent value was transformed or analyzed with the nparLD package.^40^ The main packages used were lme4 (V. 1.1–27.1),^41^ lsmeans (V. 2.30–0),^42^ multcomp (V. 1.4–18),^43^ and nparLD (V. 4.2.2).^40^ Unconstrained ordination using nonmetric multidimensional scaling (NMDS) implemented in the function metaMDS of the R package vegan version 2.6.4^44^ was used to visualize the community structures of bacteria and archaea. Differences among community compositions were assessed by PERMANOVA with a repeated measures design using PRIMER7.^45^ Relative abundances of ASVs, species, and genera (i.e., sum of ASVs assigned to the same species or genus) were correlated to gas measurements, as well as to qPCR quantifications using Pearson correlation and Benjamini-Hochberg p-value adjustments.

## Results

### Fermentation Parameters

The mean pH value during the 5 days of measurement ranged between 6.92 and 7.05, with significantly higher values (*p* < 0.001) obtained with the treatment with EA. NH_3_ formation was significantly reduced in all treatments, mainly by EA and EA+GA (−46% and −56%, respectively, *p* < 0.001) and to a lesser extent by GA (−19%, *p* < 0.001) compared to CTR. Total gas production was not significantly altered by any treatment (*p* > 0.05), but the interaction of factors treatment and time was significant (*p* < 0.01). The composition of the gas produced was affected by the treatment. Indeed, the average daily CH_4_ production was significantly reduced by EA (−46%, *p* < 0.001) and EA+GA (−60%, *p* < 0.001) compared to CTR, whereas GA had no effect (*p* > 0.05). The average daily CO_2_ production followed the same trend of CH_4_, but the reduction rate of CH_4_ was higher than that of CO_2_, as shown by the CH_4_/CO_2_ ratio (*p* < 0.001). Moreover, the average daily H_2_ production was not significantly altered by the treatments (*p* > 0.05) (Table 1).

**Table 1:** Rumen Fermentation Parameters Following Tannin Treatment^a^^b^

Parameters	CTR	GA	EA	EA+GA	SEM	*p*-value
pH	6.95^ab^	6.92^a^	7.05^c^	7.01^bc^	0.02	<0.001
NH_3_ (mmol/L)	6.1^a^	4.8^b^	3.2^c^	2.6^d^	0.3	<0.001
Gas measurements						
Total gas (ml/day)	3369	3469	3150	3301	140	0.11
CH_4_ (ml/day)	121.6^a^	106.4^a^	65.7^b^	48.9^b^	6.1	<0.001
CH_4_/VFA (mL/g)	2115^a^	1664^b^	1402^b^	890^c^	125	<0.001
CH_4_/OM (mL/g)	12.6^a^	10.3^b^	6.3^c^	4.4^c^	0.6	<0.001
CO_2_ (ml/day)	915^a^	979^a^	721^b^	784^b^	49	<0.05
CH_4_/CO_2_	0.13^a^	0.11^b^	0.09^c^	0.06^d^	0.003	<0.001
H_2_ (ml/day)	4.2	4.8	5.5	5.1	0.7	0.48
VFA						
Total VFA (mol/g)	78.4^a^	83.0^a^	58.2^b^	65.7^b^	3.83	<0.001
VFA profile						
Acetic acid (%)	49.1^a^	52.9^a^	42.8^b^	48.2^a^	1.29	<0.001
Propionic acid (%)	21.2^a^	18.3^b^	18.9^b^	15.0^c^	0.36	<0.001
Isobutyric acid (%)	0.8^a^	0.7^b^	0.6^c^	0.5^d^	0.04	<0.001
Butyric acid (%)	19.0^b^	19.5^b^	24.8^a^	26.6^a^	0.91	0.02
Isovaleric acid (%)	2.7^a^	2.6^a^	1.0^b^	0.8^c^	0.25	<0.001
Valeric acid (%)	7.3^bc^	5.9^c^	11.9^a^	9.0^b^	0.51	<0.001
Nutrient degradation						
Dry matter (%)	74.4^a^	73.8^a^	66.3^b^	66.4^b^	1.20	<0.001
Organic matter (% of DM supply)	73.0^a^	70.3^a^	61.7^b^	59.1^b^	1.39	<0.001
Crude fiber (% of DM supply)	43.4^a^	37.7^a^	19.0^b^	27.7^b^	1.91	<0.001
Neutral detergent fiber (% of DM supply)	51.5^a^	45.2^a^	29.3^b^	27.4^b^	2.22	<0.001
Acid detergent fiber (% of DM supply)	45.4^a^	39.8^a^	22.0^b^	20.9^b^	2.19	<0.001
Crude protein (% of DM supply)	88.5^a^	86.4^ab^	82.1^bc^	78.7^c^	1.37	<0.001

a Values are averages of the whole sampling period (days 6–10).

b Abbreviations: CTR: control; EA: ellagic acid; GA: gallic acid; SEM: standard error of the means. Means with different superscripts within a row are significantly different (*p* < 0.05). ^a,b,c^Least square means with different superscripts differ (*p* < 0.05).

Compared to CTR and GA, the EA and EA+GA treatments significantly (*p* < 0.001) reduced total VFA production. The average reductions in VFA production were 26% (EA) and 16% (EA+GA) relative to CTR. The reduced total VFA was also associated with an altered VFA profile. Acetic acid was significantly lowered only by EA (−13%, *p* < 0.001), propionic acid was significantly decreased by all treatments (*p* < 0.001), and most VFA by EA+GA (−29%), whereas butyric acid was significantly increased by both EA and EA+GA (*p* < 0.05, + 30% and +40%, respectively) (Table 1).

The altered gas and VFA production were also related to an altered rate of nutrient degradation, because EA and EA+GA treatments affected feed fermentation by significantly reducing the degradation of nutrients (*p* < 0.001). Considering the composition of the feed substrate, the fiber degradation is of notable interest because EA and EA+GA reduced the degradation of CF (−56% and −36%, respectively), NDF (−43% and −47%, respectively), and ADF (−52% and −54%, respectively) more than the other feed components measured. Instead, no significant change was caused by the addition of GA (Table 1).

**Figure 1:**
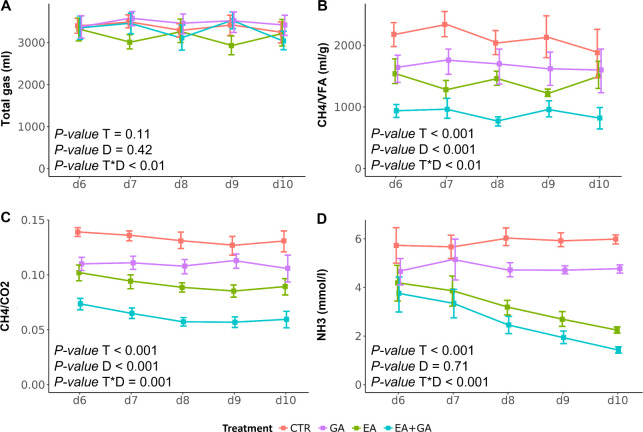
Kinetics of total gas production (A), CH4/VFA (B), CH4/CO2 (C), and NH3 (D) during the sampling period (days 6–10). The daily addition of GA and EA lasted from day 1 to day 10. Abbreviations: *T* = treatment; D = day. Error bars represent the standard error.

Parameters that revealed variable treatment effects over time (i.e., significant interaction effect of treatment and time) included total gas, CH_4_/VFA, CH_4_/CO_2_ and NH_3_ (Figure 1A–D). For total gas (Figure 1A), the EA treatment at day 9 had lower (*p* < 0.05) values compared to CTR and GA at day 7, GA and EA+GA at day 9. An evident interaction of treatment and time factors was found for all other parameters and especially for NH_3_ production, for which it is possible to observe a decreasing production over time in EA and EA+GA groups (Figure 1D). Specifically, in comparison to the control, the treatments EA and EA+GA resulted in an increasing and significant (*p* < 0.05) reduction of NH_3_ from day 6 to day 10 (Figure 1D).

### Urolithins

**Figure 2:**
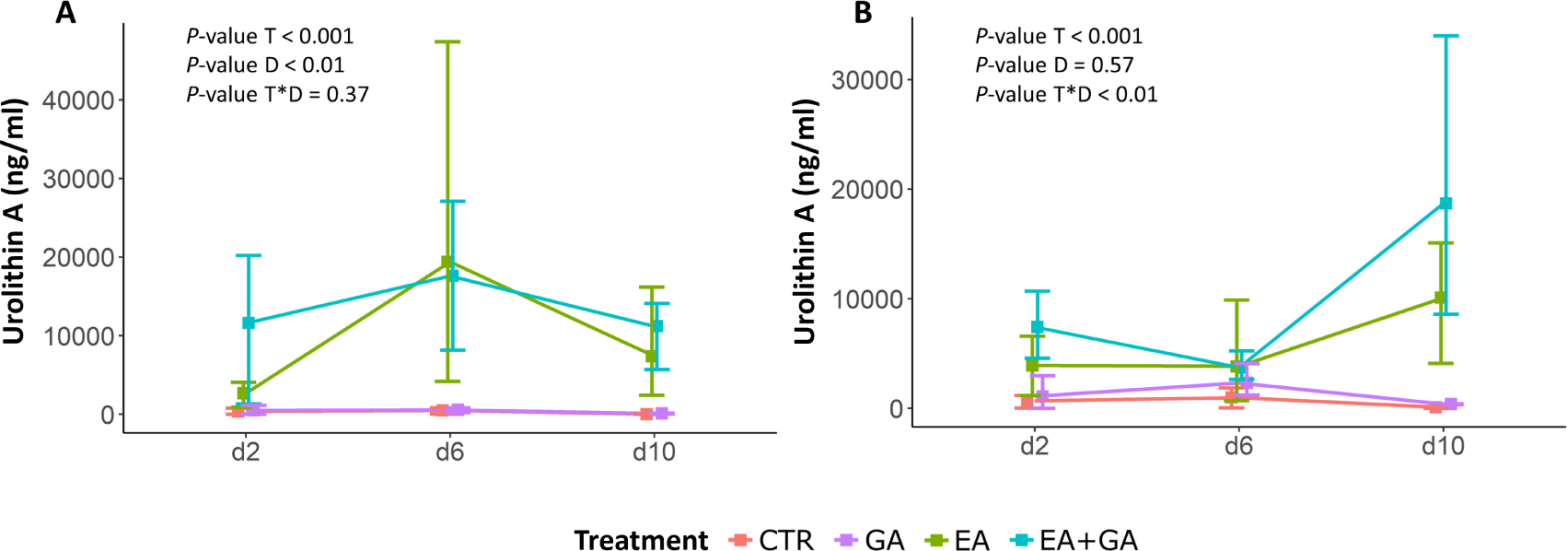
Level of UroA (A) and UroB (B) in the incubation fluid at days 2 (d2), 6 (d6), and 10 (d10) of in vitro fermentation. The daily addition of GA and EA lasted from day 1 to day 10. Abbreviations: CTR = control; GA = gallic acid; EA = ellagic acid; *T* = treatment; D = day. Error bars represent the standard error.

UroA and UroB levels in rumen fluid were significantly modulated by the EA and EA+GA treatments (*p* < 0.001), whereas GA did not exert any modulating effect in any case (*p* > 0.05). Specifically, EA and EA+GA significantly increased UroA compared to CTR at day 6 (both *p* < 0.001 vs CTR) and day 10 (both *p* < 0.001 vs CTR, Figure 2A). For UroB, the only significant difference was observed at day 10, when EA and EA+GA significantly increased UroB (*p* < 0.05 and *p* < 0.01, respectively) compared to CTR and GA (Figure 2B).

### Protozoan and Bacterial Counts

**Figure 3:**
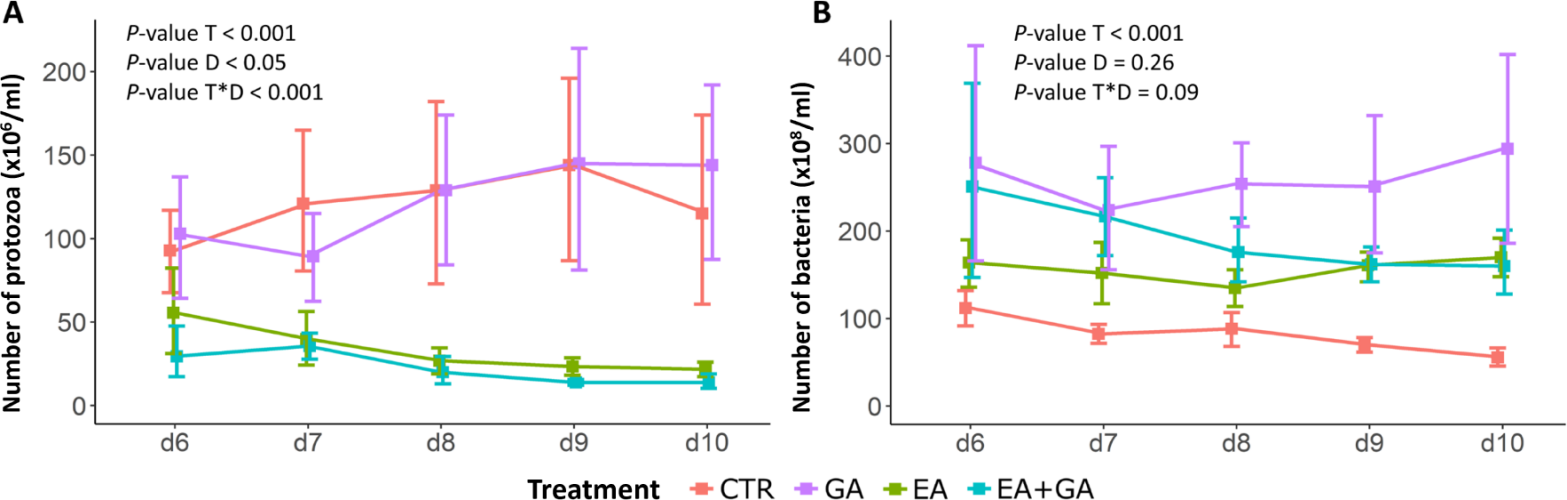
Number of protozoa (A) and bacteria (B) during the sampling period (days 6–10). The daily addition of GA and EA lasted from day 1 to day 10. Abbreviations: CTR = control; GA = gallic acid; EA = ellagic acid; *T* = treatment; D = day. Error bars represent the standard error.

The number of protozoa was significantly decreased by EA and EA+GA (*p* < 0.001), whereas it did not differ between GA and CTR (*p* > 0.05) (Figure 3A). The difference in protozoa counts increased over time among the treatments with and without EA (*p* < 0.05). From day 8 onward, the number of protozoa following EA and EA+GA treatments was significantly lower than the number of protozoa following CTR and GA (*p* < 0.001). Conversely, the number of bacteria was lowest in CTR, and remained stable over days 6–10 (*p* > 0.05) (Figure 3B). In particular, GA (*p* < 0.001) and EA (*p* < 0.05) significantly increased bacterial counts in comparison to CTR, whereas EA+GA only showed an increasing tendency over CTR (*p =* 0.053). Further, the number of bacteria was higher in GA treatments as compared to EA (*p* < 0.05) and EA+GA (*p* < 0.01).

### Bacterial and Archaeal Quantification Through Real-Time qPCR

The EA and GA treatments differently modulated the abundance of a panel of five bacterial species, one bacterial group (*Prevotella*) involved in rumen fermentation, and one archaeal genus (*Methanobrevibacter*). Both EA and EA+GA decreased the relative abundance of *Butyrivibrio fibrisolvens* (*p* < 0.001), *Fibrobacter succinogenes* (*p* < 0.001), and *Ruminococcus flavefaciens* (*p* < 0.001) compared to CTR, considering the same time points (day 6 and day 10), with the only exception of *Ruminococcus flavefaciens* at day 6 for EA, EA+GA, and CTR (*p* > 0.05). The abundance of *Selenomonas ruminantium* was significantly increased only by EA+GA (*p* < 0.001) at day 6 and day 10 compared to CTR. For archaeal *Methanobrevibacter*, a significant difference was observed at day 6, when EA significantly increased levels of *Methanobrevibacter* (*p* < 0.01) compared to CTR and GA (Table 2).

**Table 2:** Relative Abundance of Selected Rumen Bacteria and Archaea Following Tannin Treatments on Day 2 (d2), Day 6 (d6), and Day 10 (d10)^a^

	**CTR**			**GA**			**EA**			**EA+GA**			**SEM**	*p***-value**		
Target^b^	**d2**	**d6**	**d10**	**d2**	**d6**	**d10**	**d2**	**d6**	**d10**	**d2**	**d6**	**d10**		**T**	**D**	T*D
*B. fibr*	0.39^a^	0.30^a^	0.35^a^	0.39^a^	0.15^abc^	0.20^abc^	0.3 ^ab^	0.08^bc^	0.01^c^	0.32^ab^	0.09^bc^	0.01^c^	0.07	<0.001	<0.001	<0.01
*F. succ*	0.23^a^	0.52^a^	0.20^a^	0.26^a^	0.36^a^	0.13^a^	0.22^a^	0.01^b^	<0.001^c^	0.21^a^	0.003^b^	<0.001^c^	0.03	<0.001	0.052	<0.001
*R. alb*	0.22	2.13	1.18	0.24	3.69	1.44	0.26	0.65	1.81	0.20	0.87	2.90	0.77	0.08	<0.01	0.25
*R. flav*	0.27^ab^	0.17^bcde^	0.20^bcd^	0.31^a^	0.15^cde^	0.13^de^	0.31^a^	0.11^def^	0.08^ef^	0.25^abc^	0.09^def^	0.005^f^	0.02	<0.001	<0.01	<0.01
*S. rum*	8.93^d^	20.10^cd^	21.10^cd^	8.92^d^	20.40^cd^	24.60^bcd^	11.30^d^	42.80^abc^	40.60^abc^	8.45^d^	57.20^a^	46.40^ab^	3.37	<0.001	0.13	<0.001
*Prevotella*	1.09	0.67	0.44	1.05	0.78	0.67	1.08	0.95	0.52	1.09	0.92	0.55	0.09	0.32	<0.001	0.39
*Methanobrev*	2.51^abc^	1.16^bc^	1.69^abc^	3.26^a^	1.12^c^	1.53^abc^	3.10^ab^	3.33^a^	2.84^ab^	2.76^abc^	1.17^abc^	1.74^abc^	0.72	<0.01	<0.01	<0.05

a Abbreviations: CTR = control, GA = gallic acid, EA = ellagic acid, SEM = standard error of the mean, *T* = treatment, D = day. Means with different superscripts within a row are significantly different (*p* < 0.05). ^a,b,c^Least square means with different superscripts differ (*p* < 0.05).

b Bacteria: B. fibr (Butyrivibrio fibrisolvens), F. succ (Fibrobacter succinogenes), R. alb (Ruminococcus albus), R. flav (Ruminococcus flavefaciens), S. rum (Selenomonas ruminantium); Archaea: Methanobrev (Methanobrevibacter).

### Bacterial and Archaeal Community Analyses Based on Metabarcoding

**Figure 4:**
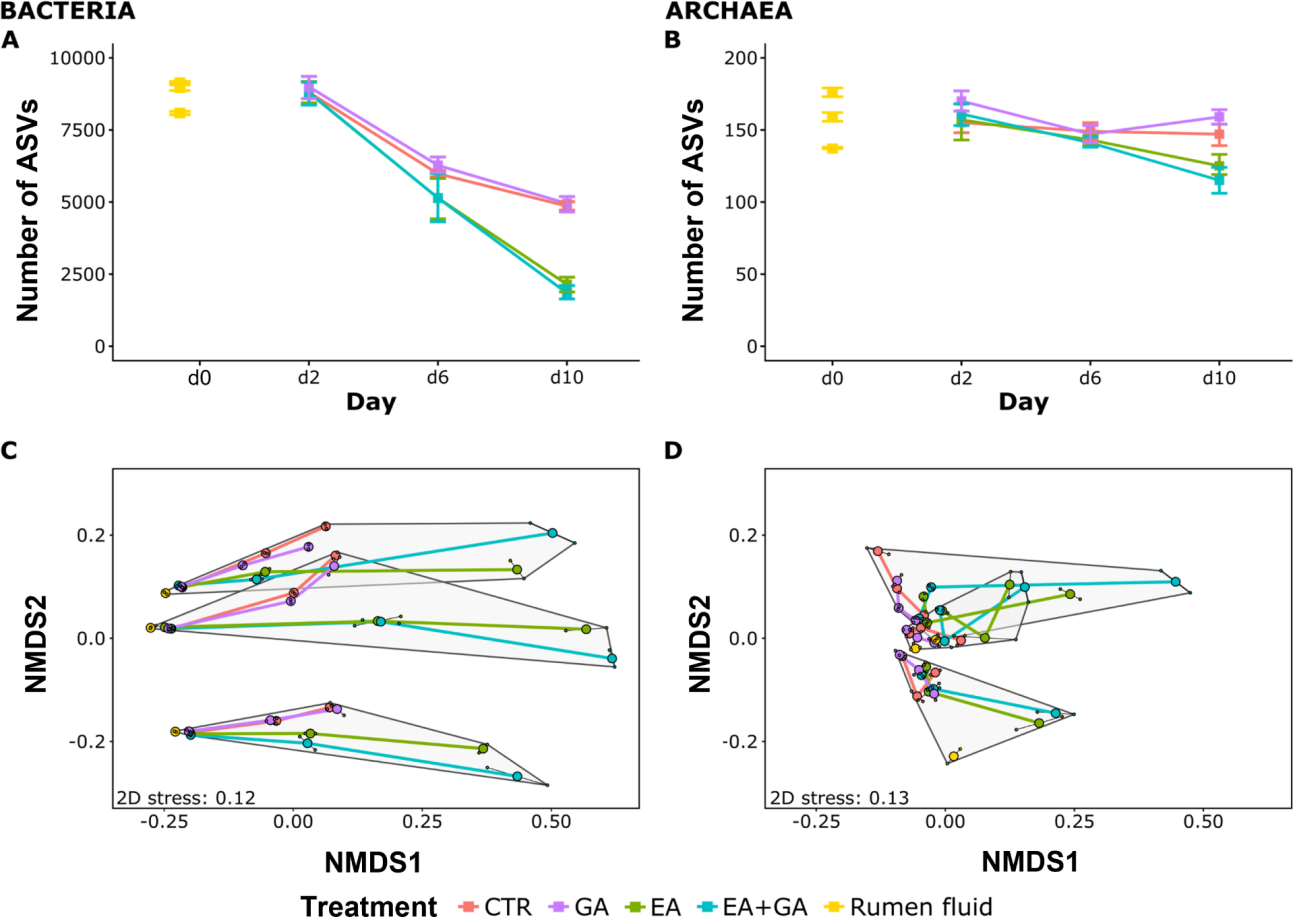
Bacterial ASV richness (A), archaeal ASV richness (B) on day 2 (d2), day 6 (d6), and day 10 (d10) of Rusitec and community compositions of bacteria (C) and archaea (D). Baseline parameters of the “Rumen fluid” samples from the three runs are reported on day 0 (d0) in Figure 4A and 4B. In Figure 4C and 4D, the lines connect the means of two samples from the same experimental run. Community compositions are visualized by nonmetric multidimensional scaling (NMDS) based on Jaccard similarities. Gray polygons regroup samples from the same experimental run. Small black dots linked to the means by thin lines indicate the measured samples. ASV richness and Jaccard similarities are the means of 1,000 iterative subsamples of raw communities to the lowest read number of a sample. Abbreviations: CTR = control, EA = ellagic acid, GA = gallic acid. Error bars represent the standard error.

A total of 17,836 bacterial and 274 archaeal ASVs were detected. A classification to species level was possible for 6584 bacterial ASVs (669 species, 1–321 ASVs per species) and 110 archaeal ASVs (18 species, 1–32 ASVs per species). The other ASVs could only be classified to a higher taxonomic level. Bacterial ASV richness, evenness, and diversity significantly decreased over time in all treatments (*p* < 0.001), but a stronger decrease was observed following EA and EA+GA as compared to CTR and GA (*p* < 0.001, Figures 4A and S1). Archaeal ASV richness was significantly decreased by EA and EA+GA from day 2 to day 10 (*p* < 0.001), and at day 10 EA and EA+GA were significantly lower than the CTR and GA (*p* < 0.01, Figure 4B). However, the decrease in ASV richness from day 2 to 10 following all treatments was larger for bacteria (−60% on average) as compared to archaea (−15% on average). In contrast to bacteria, archaeal ASV evenness and diversity were not significantly affected by treatments and time (*p* > 0.05, Figure S1).

Bacterial and archaeal community compositions differed significantly among the three experimental runs (Figure 4C,D), revealing differences among the communities established in the Rusitec system, possibly due to the initially sampled rumen fluids from the donor animals. On average, 46% of bacterial and 75% of archaeal ASVs were shared among samples from different rumen fluids. Despite some differences in rumen fluids, EA and EA+GA caused a consistent community shift along the same ordination axis (NMDS1, Figure 4C). A similar effect of EA addition was also detected on archaeal communities that shifted along the first ordination axis (Figure 4D). Overall, treatment, time, and run had a significant effect on bacterial and archaeal communities (PERMANOVA, *p* < 0.05).

**Figure 5:**
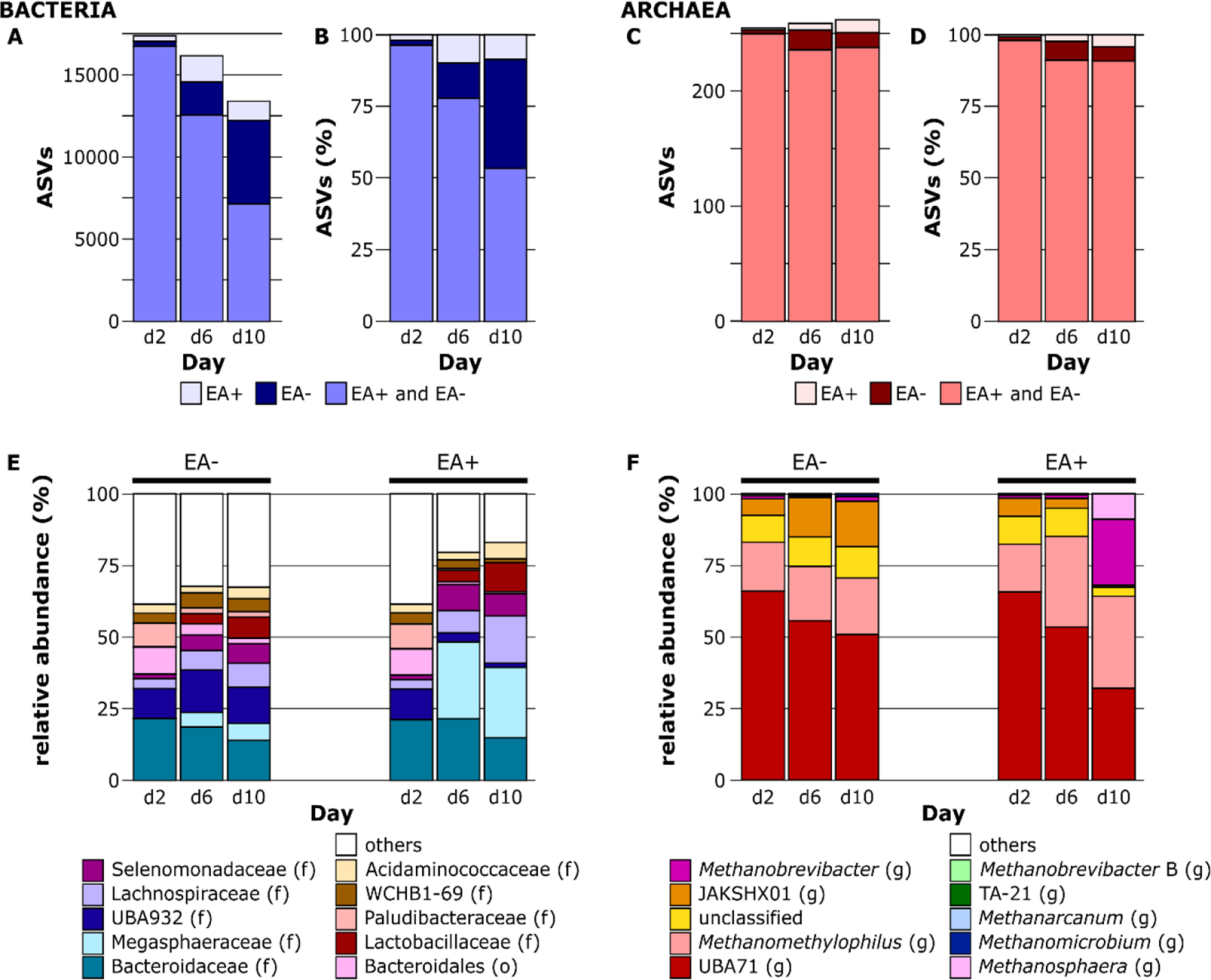
Differences in microbial community compositions of treatments with EA (EA+) or without (EA-). Bacterial (A, B) and archaeal (C, D) ASVs detected only in samples with EA (light blue/red), detected in samples with and without EA (blue/red), and detected only in samples without EA (dark blue/red). The lower panels show the relative abundances of the ten most abundant bacterial families (E) and archaeal genera (F). Mean relative abundances of samples with (EA+) or without (EA-) EA are shown per day (2, 6, and 10). Less abundant families and genera are summed in the category “others” (white). Letters in parentheses indicate the level of taxonomic classification, that is, genus (g), family (f), or order (o).

To better understand the community shifts related to the addition of EA, we compared EA and EA+GA (EA+) to CTR and GA (EA-) treatments in greater detail. The number of shared bacterial ASVs among EA+ and EA- decreased from 96.3% by day 2 to 53.4% by day 10 (Figure 5B), whereas a smaller decrease from 98.0% to 90.8% was found for archaea (Figure 5D). In parallel with the decrease in shared bacterial ASVs among EA+ and EA-, we observed an increase in bacterial ASVs that were detected in EA- only, from 1.7% by day 2 to 12.4% by day 6 and 37.9% by day 10 (Figure 5B). This suggests that many of the observed bacterial species were sensitive to EA. However, EA application also led to an increased relative abundance of some bacterial ASVs, which was strongest for ASVs classified into the Megasphaeraceae family. Other minor alterations in the bacterial communities were the decrease in the candidate genus UBA932 (family Bacteroidaceae) and the increase in the family Lachnosphiraceae in EA+ (Figure 5E). The taxonomic composition of archaeal communities was characterized by a decrease in the most abundant candidate genus UBA71 (family Methanomethylophilaceae) in all treatments over time, as well as by an increase in the candidate genus JAKSHX01 (also family Methanomethylophilaceae) in EA- treatments. The candidate genus JAKSHX01 was negatively affected by the EA+ treatments, disappearing by day 10. By contrast, the archaeal genera *Methanomethylophilus* and *Methanosphaera* showed increased abundance following EA+ treatments. Furthermore, *Methanobrevibacter* became much more abundant in EA+ by day 10 (23.0%) compared to days 2 (1.0%) and 6 (1.2%) (Figure 5F).

### Comparison of qPCR and High-Throughput Sequencing

With the exception of *B. fibrisolvens*, all taxa quantified by qPCR were represented by multiple ASVs obtained by high-throughput sequencing. The number of ASVs within a taxon targeted by qPCR ranged from 2 ASVs (*Ruminococcus albus*) to 1362 ASVs (*Prevotella*). Correlations of qPCR values with summed relative abundances obtained by high-throughput sequencing varied widely, ranging from −0.33 to 0.98. Insignificant, negative, or weak correlations below 0.6 were obtained for *Butyrivibrio fibrisolvens* (*r* = −0.33, *p =* 0.004), *Selenomonas ruminantium* (*r* = 0.17, *p =* 0.146), and *Methanobrevibacter* (*r* = 0.55, *p* < 0.0001). Strong correlations were obtained for *Ruminococcus flavefaciens* (*r* = 0.73, *p* < 0.0001), *Prevotella* (*r* = 0.80, *p* < 0.0001), *Fibrobacter succinogenes* (*r* = 0.93, *p* < 0.0001), and *Ruminococcus albus* (*r* = 0.98, *p* < 0.0001).

### Correlations of Bacterial and Archaeal ASVs and Species to CH_4_ Production

**Figure 6:**
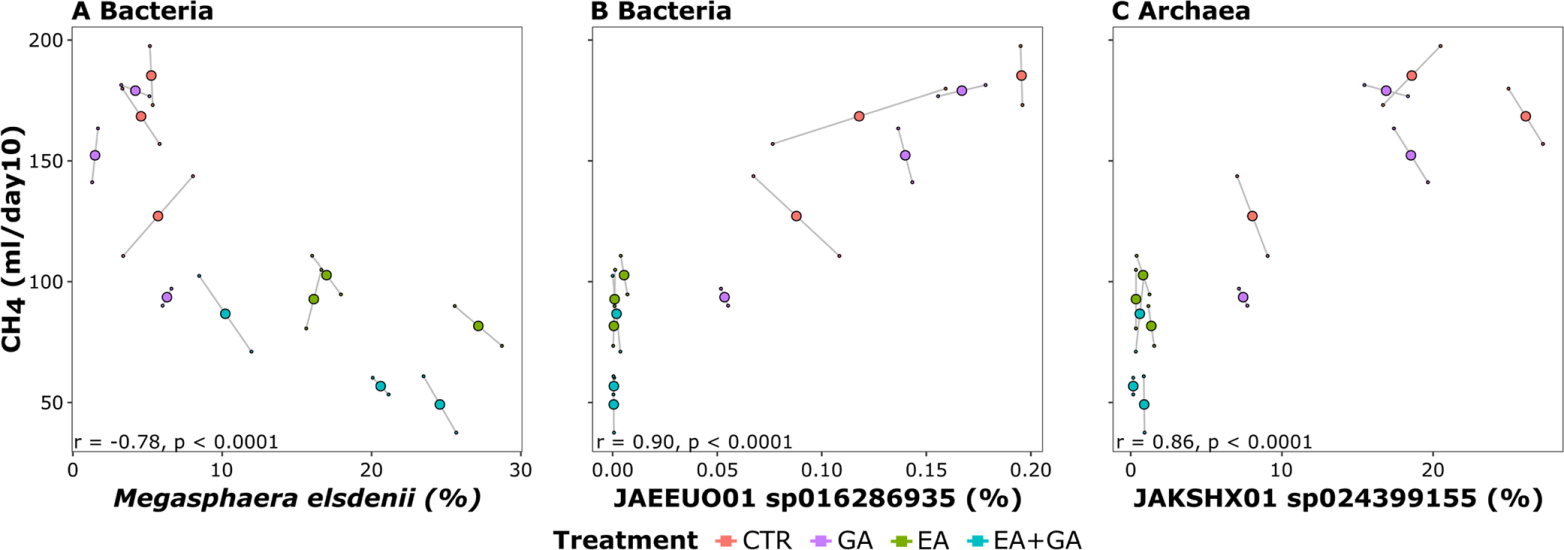
Correlations of relative abundances of species to CH_4_ emissions at day 10. Bacterial species with the strongest negative (A) and positive (B) correlations and archaeal species with the strongest positive (C) correlations are shown. Colors indicate treatments, that is, control (CTR), gallic acid (GA), ellagic acid (EA), and their combination (EA+GA). Larger points indicate averages of two replicates indicated as smaller connected points.

To identify potential links between microbial communities and CH_4_ production, correlation analyses of the relative abundances of bacterial and archaeal ASVs and CH_4_ emissions on day 10 were performed. Among the 13,421 detected bacterial ASVs and 262 detected archaeal ASVs on day 10, 35 bacterial and 8 archaeal ASVs were positively correlated to CH_4_ emissions on day 10, while 3 bacterial and no archaeal ASVs were negatively correlated to CH_4_ emissions on day 10 (*P*. adjusted <0.05, |r| > 0.7). The three bacterial ASVs that were strongly and negatively correlated to CH_4_ emissions were two ASVs classified into the genus *Megasphaera* (both *r* = −0.73, *p* < 0.0001), of which one could be classified as the species *M. elsdenii*, and one ASV classified into the family Lachnospiraceae without genus or species assignment (*r* = −0.71, *p* < 0.0001). Relative abundances summed at the species level also showed *M. elsdenii* with the strongest negative correlation with CH_4_ emissions (*r* = −0.78, *p* < 0.0001, Figure 6A). *M. elsdenii* was the dominant bacterial species in both EA- and EA+, but its relative abundance at day 10 was 19.3%, much higher in EA+ than in EA- (4.6%). The strongest positive correlations of bacterial and archaeal species with CH_4_ emissions on day 10 were detected for GTDB candidate species JAEEUO01 sp016286935 of the bacterial family Anaerovoracaceae (*r* = 0.90, *p* < 0.0001, Figure 6B) and GTDB candidate species JAKSHX01 sp024399155 of the archaeal family Methanomethylophilaceae (*r* = 0.86, *p* < 0.0001, Figure 6C).

## Discussion

The Rusitec system allowed us to study the activity of two individual HT subunits, alone and in combination, in rumen and over 10 days of continuous and standardized fermentation. The current outcomes were similar to what observed after a 24-h Hohenheim gas test trial.^26^ However, the coupling of Rusitec with high-throughput sequencing of two marker genes enabled us to observe the kinetics of the fermentation parameters of interest and of associated alterations in bacterial and archaeal community compositions over 10 days. We acknowledge that the dosages of EA and GA used in this study were higher than what would typically be applied in in vivo scenarios. However, our primary objective was to investigate and elucidate how HT affect gas emissions and the rumen microbiota under controlled in vitro conditions.

The daily production of CH_4_ and CO_2_ per ml of total gas was significantly reduced by EA and EA+GA but not by GA alone. However, total gas and daily H_2_ production per ml of total gas were not significantly altered by the treatments (Table 1). Our findings on GA used at 75 mg/g DM are consistent with those of Wei et al.^46^ who used GA up to 20 mg/g DM in a Rusitec fermentation model and observed no variation in total gas, daily CH_4_, and H_2_ production. The effect of tannins on CH_4_ emission, in particular EA, could be a consequence of direct alterations of the archaeal methanogen community or of changes in communities of other microorganisms that affect the CH_4_ production, for instance, by reducing substrates needed for methanogenesis. Here, a treatment effect was more evident on bacterial diversity than on archaeal diversity and community compositions. Based on qPCR, a reduction in the relative abundances of *B. fibrisolvens*, *F. succinogenes*, and *R. flavefaciens* was observed following EA addition. These organisms produce H_2_ during feed fermentation, and H_2_ and CO_2_ are the building blocks used by hydrogenotrophic methanogens to produce CH_4_.^47^ In parallel, EA addition increased the relative abundance of *S. ruminantium*, a nitrate-reducing bacterial species able to grow on tannic acid or CTs,^48^ and compete with methanogens because nitrate is considered an alternative H_2_ sink,^49^ although a correlation between tannins and nitrate levels was not determined in this study. Furthermore, *Prevotella* is known to be one of the most dominant genera in rumen microbiomes,^50^ and its abundance was reported to be inversely correlated with CH_4_ emissions.^51^ However, in our experiment, we observed a strong reduction of *Prevotella* relative abundances from days 2 to 10 (see Table 2), while smaller differences were found among treatments. In Manoni et al.^26^ an increased relative abundance of *Prevotella* was reported following EA treatment at 150 mg/g, while EA and GA treatments at 75 mg/g did not result in a significant increase of *Prevotella* abundances. In another *in vivo* trial, a reduced abundance of Prevotella (−5.4%) was reported in Holstein cows fed a mixture of quebracho and chestnut tannins,^52^ thus indicating that the influence of tannins on *Prevotella* may depend on the specific tannin molecules. The reduction of Prevotella abundance across both control and treatment groups in our study may indicate that the unique conditions of the Rusitec system contributed to this effect, independent by the tannin exposure. Further studies are needed to clarify how the in vitro conditions of the Rusitec system affect the Prevotella population compared to in vivo conditions.

Furthermore, high-throughput sequencing showed that EA and EA+GA increased the relative abundances of the bacterial families Lachnospiraceae and Megasphaeraceae, both negatively correlated with CH_4_ production. Lachnospiraceae is positively correlated with butyrate production,^53^ as consistently observed following EA and EA+GA treatments. Regarding other VFA, EA decreased both acetate and propionate, whereas EA+GA only reduced propionate. Acetate and butyrate are associated with H_2_ production, while propionate is associated with H_2_ consumption.^54,55^ The alteration of the rumen microbial community may have influenced the VFA profile and in turn H_2_ production. Given the low effects of GA, the four treatment groups were further categorized into EA+ (containing EA) and EA- (lacking EA). Such categorization allowed us to further differentiate the effects of EA on fermentation and microbial community structures. The bacterial family Megasphaeraceae, particularly sequences associated with the species *M. elsdenii*, were strongly increased by EA+ treatments at day 10 (compared to EA- treatments) and showed the highest negative correlation with CH_4_ production. *M. elsdenii* is a lactate-consuming species that has already been correlated with lower rumen CH_4_ production.^21,56,57^ The action of *M. elsdenii* on CH_4_ mitigation can be likely explained by the conversion of lactate to propionate, which works as an alternative H_2_ sink that subtracts H_2_ from CH_4_ production.^58^ In this study, propionate was not increased by the treatments. However, in long-term fermentation simulation techniques, such as Rusitec, *M. elsdenii* reportedly utilizes H_2_ to convert lactate to pyruvate instead of propionate by an NAD-independent lactate dehydrogenase enzyme.^21,59,60^ Therefore, the observed CH_4_ reduction could be ascribed to a redirection of H_2_ to an alternative H_2_ sink that is not propionate but pyruvate.

Archaeal communities were less affected by the addition of GA and EA, in agreement with previous findings reporting that rumen archaea are less prone to compositional variations than bacteria across ruminant species.^50^ The archaeal community was dominated by the Methanomethylophilaceae family following all treatments. Methanomethylophilaceae are methylotrophic archaea and part of methanogens commonly found in the rumen.^29,50^ Methylotrophic methanogens produce CH_4_ using H_2_ to reduce methylated compounds, such as methanol.^21,29^ The candidate genus JAKSHX01 of Methanomethylophilaceae had the strongest positive correlation with CH_4_ production and was reduced by EA+ treatments. EA+ treatments also increased the hydrogenotrophic archaeal genera *Methanosphaera* and *Methanobrevibacter*, although EA+ treatments were effective in reducing CH_4_. Another study evaluating the effect of various sources of HT and CT plant extracts on the modulation of rumen microbiota showed that *Methanobrevibacter* was not reduced by any tannin source or supplementation dose,^61^ suggesting that only the metabolic activity but not the abundance of *Methanobrevibacter* was affected by the EA+ treatments. In any case, the cause–effect relationship between reduced ruminal CH_4_ emission and relative abundances of methanogens remains to be demonstrated, because CH_4_ emissions can be reduced even without reducing the abundance of methanogens.^62^ Another factor to be considered is the low versus high CH_4_-yield emission phenotype of the animals, given by the microbial composition and the number of methanogenesis-related gene transcripts.^63^

The EA+ treatments reduced protozoa, known to indirectly support CH_4_ emissions through H_2_ production.^10^ Protozoa depletion has been linearly correlated with reduced protein degradation and NH_3_ formation.^64−66^ Tannins can interfere with protein degradation and NH_3_ formation, as tannins bind proteins to form macromolecular complexes that reduce protein availability for microbial degradation in the rumen. This complex formation protects proteins from microbial degradation in the rumen resulting in increased protein amounts reaching the host abomasum and the small intestine, where they are absorbed. As a consequence, urinary nitrogen excretion and N_2_O emissions are decreased.^10,67^ The progressive decreasing trend in NH_3_ formation observed from day 6 to day 10 led us to hypothesize that the effects of EA on the rumen microbiota became more pronounced on day 10 than on day 6, possibly disadvantaging protozoa and protein-degrading bacteria despite the daily addition of fresh feed material. Although reduced protein degradation can increase intestinal absorption of proteins, CP degradation still remains a concern regarding tannin effects in rumen in the *in vivo* condition. Along with CP, OM and NDF degradation were reduced as well by EA+ treatments. Other explanations for the reduced nutrient degradation are the lower bacterial richness, which may be related to a reduced diversity of bacterial enzymes for nutrient degradation, and the reduced relative abundance of bacteria able to ferment fibers and cellulose at the rumen level, such as *B. fibrisolvens*, *F. succinogenes*, and *R. flavefaciens*. Finally, the amount of substrate per unit of fermentation medium volume, which is smaller than the *in vivo* condition, could have affected the results.^68^ The use of tannins and the lower degradation of nutrients is similar to other studies on other sources of tannin compounds such as chestnut extract,^69^ fibrous feed sources such as brachiaria, beet, and apple pomace,^70^ feed concentrates such as barley and soybean meal,^71^ choline,^21^ and a blend of essential oils and plant tannin extracts.^22^

As reported above, urolithins are secondary metabolites produced by the bacterial degradation of ellagitannins.^26,72^ The conversion of Iso-UroA to UroB seems to be favored as compared to the conversion of UroA to UroB,^23,24^ although this latter metabolic pathway has also been suggested.^25,73^ Unfortunately, in our study we did not quantify the Iso-UroA and this aspect cannot be clarified. However, for the first time, we characterized UroA and UroB in rumen fluid in a Rusitec system, revealing increased UroA concentrations as early as day 6. We speculate that specific tannin-degrading bacteria, active during the experimental period of 10 days, may have produced UroA that was further converted to UroB. However, due to the current lack of studies focusing on urolithin concentrations in rumen fluids and Rusitec systems, further research is needed to unravel the metabolism of tannin-degrading and urolithin-producing bacteria.

Overall, EA and EA+GA showed a stronger impact compared to GA alone, suggesting that EA is the primary contributor to the observed effects, with no additive effect from the combination of EA and GA. EA addition altered the bacterial diversity and decreased the archaeal diversity and community compositions, consequently driving the related outcomes of rumen fermentation, such as CH_4_, VFA, NH_3_, and nutrient degradation. GA addition resulted in fewer detrimental effects on VFA production and nutrient degradation than EA. UroA and UroB were quantified for the first time in rumen fluid following Rusitec, although the complete metabolic pathway has not yet been fully clarified. The sum of increased abundance of *M. elsdenii*, decreased abundance of Methanomethylophilaceae, protozoa depletion, and lower bacterial richness over time may explain the observed CH_4_ decrease and the related effects following EA and EA+GA treatments. To conclude, EA-containing plant extracts could be applied as effective dietary supplements for reducing enteric methane production, even though some negative effects on rumen fermentation were observed, and more information is needed before a potential *in vivo* application.

## Supporting Information Available

Bacterial ASV evenness (A) and diversity (B) and archaeal ASV evenness (C) and diversity (D) on day 2 (d2), day 6 (d6), and day 10 (d10) of Rusitec following tannin treatments (PDF)

## Abbreviations

ADF: acid detergent fiber
ADL: acid detergent lignin
ANOVA: analysis of variance
ASV: amplicon sequence variant
CF: crude fiber
CH_4_: methane
CO_2_: carbon dioxide
CP: crude protein
CT: condensed tannins
CTR: control
DM: dry matter
dOM: digestible organic matter
EA: ellagic acid
EA+GA: combination of EA and GA
EE: ether extracts
GA: gallic acid
GHG: greenhouse gases
GTDB: genome taxonomy database
H_2_: hydrogen
HT: hydrolyzable tannins
NDF: neutral detergent fiber
NH_3_: ammonia
NMDS: nonmetric multidimensional scaling
OM: organic matter
PERMANOVA: permutational multivariate analysis of variance
qPCR: quantitative polymerase chain reaction
Rusitec: rumen simulation technique
VFA: volatile fatty acids
SEM: standard error of the means
TMR: total mixed ration
UPLC-HRMS: ultraperformance liquid chromatography–high-resolution mass spectrometry
UroA: urolithin A
UroB: urolithin B
