# Supplementary material for: Gallic and Ellagic Acids Differentially Affect Microbial Community Structures and Methane Emission When Using a Rumen Simulation Technique
Source: J Agric Food Chem. 2024 Nov 26;72(49):27163–76. doi: 10.1021/acs.jafc.4c06214 (PMC11638960; doi:10.1021/acs.jafc.4c06214)
Supplement: Supplementary file 1 — jf4c06214_si_001.pdf [file jf4c06214_si_001.pdf]

Supporting Information

Gallic and ellagic acids differentially affect microbial community structures and methane emission when using a rumen simulation technique

Michele Manoni[a], Florian Gschwend[b], Sergej Amelchanka[c], Melissa Terranova[c], Luciano Pinotti[ad], Franco Widmer[b], Paolo Silacci[e], Marco Tretola[f]*

[a]Department of Veterinary Medicine and Animal Science, University of Milan, Via dell'Università 6, 26900 Lodi, Italy

[b]Molecular Ecology, Agroscope, 8046 Zurich, Switzerland

[c]AgroVet-Strickhof, ETH Zurich, 8315 Lindau, Switzerland

[d]CRC Innovation For Well-Being And Environment (I-WE), University of Milan, 20134 Milan, Italy

[e]Paolo Silacci – Animal Biology, Agroscope, 1725 Posieux, Switzerland

[f]Swine Research Group, Agroscope, 1725 Posieux, Switzerland; Agroscope, Swine Research Group, 1725 Posieux, Switzerland;

*Email: marco.tretola@agroscope.admin.ch

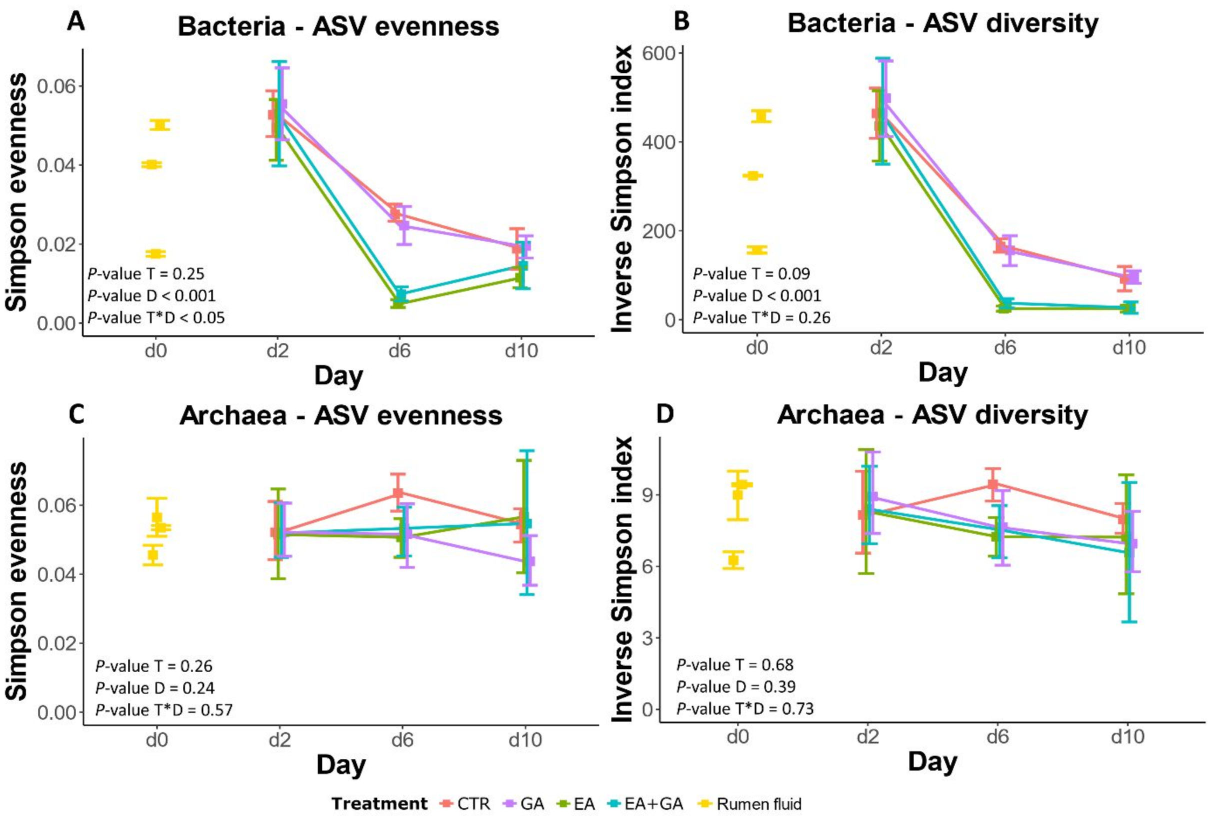

Figure S1. Bacterial ASV evenness (A) and diversity (B) and archaeal ASV evenness (C) and diversity (D) on day 2 (d2), day 6 (d6), and day 10 (d10) of Rusitec following tannin treatments. Baseline parameters of "Rumen fluid" samples from the three runs are reported on day 0 (d0). Abbreviations: CTR = control, GA = gallic acid, EA = ellagic acid, T = treatment, D = day. P-values are related only to treatments from d2 to d10 (TIF).
